# Diagnostic accuracy of ultrasound-guided core needle biopsy versus incisional biopsy in soft tissue sarcoma: an institutional experience

**DOI:** 10.1038/s41598-021-96953-w

**Published:** 2021-09-08

**Authors:** Miroslava Cernakova, Gerhard M. Hobusch, Gabriele Amann, Philipp T. Funovics, Reinhard Windhager, Joannis Panotopoulos

**Affiliations:** 1 Department of Orthopaedic Surgery, Medical University of Vienna, Vienna General Hospital, Währinger Gürtel 18-20, 1090 Vienna, Austria; 2 Clinical Institute for Pathology, Medical University of Vienna, Vienna, Austria

**Keywords:** Sarcoma, Surgical oncology

## Abstract

Core needle biopsy (CNB) is gaining in importance due to its advantages in the matter of patient morbidity, time and cost. Nevertheless, controversies still exist regarding the biopsy technique of choice for the accurate diagnosis of soft tissue sarcoma (STS). This retrospective cohort study compared the diagnostic performance between ultrasound-guided CNB and incisional biopsy (IB), both performed by orthopedic surgeons. The aims of the study were to answer the following questions: (1) Is ultrasound-guided CNB a highly reliable modality for diagnosing STSs? (2) Is CNB equally useful to IB for identifying histologic subtype? (3) Had patients who underwent CNB a reduced risk of complications? One-hundred and fifty-three patients who underwent resection of soft tissue sarcoma were classified into two groups according to biopsy technique prior to surgery; CNB group (n = 95) and IB group (n = 58). The final surgical specimens were in 40 patients liposarcoma (myxoid, pleomorphic and dedifferentiated), 39 undifferentiated pleomorphic sarcoma (UPS), 33 myxofibrosarcoma, 10 synovial sarcoma, 10 leiomyosarcoma and in the remaining 21 patients different soft tissue sarcoma entities. Sarcoma location of 71 patients was in the thigh, 19 in the lower leg, 22 in the upper arm and shoulder area; 10 in the knee and gluteal region, 9 in the thoracic region, the residual 12 in other body areas. Malignancy was correctly diagnosed in 87% (83 of 95) for the CNB group and 93% (54/58) for the IB group. Correct identification rate of histologic subtype was 80% (76 of 95) in the CNB group and 83% (48 of 58) in the IB group. There were no significant differences in the correct diagnosis rates of malignancy and subtype between the two techniques. No complications were seen in the CNB group, whereas 2 patients in whom IB was performed developed pulmonary embolism and 1 patient surgical site infection. Ultrasound-guided CNB is highly accurate and not inferior to IB in diagnosing the dignity of lesions and histologic subtype in patients with suspected STSs.

## Introduction

Soft tissue sarcomas (STS) represent a very heterogeneous group of malignant mesenchymal neoplasms with more than 50 histological subtypes^1^. While STS account for roughly only 1% of all adult malignancies^2^, there are even so over 27,000 new cases per year in EU27 countries^3^. Most STSs present in the extremities. The overall frequency of locations is thigh, buttock, and groin, 46%; torso, 18%; upper extremity, 13%; retroperitoneal, 13%; and head and neck, 9%^4^. Diagnosis is often delayed by the fact that STSs can grow to a large extent until they cause discomfort and lead to consultation, especially in the thigh or pelvic regions^5,6^.

“Even for clinically unequivocal sarcomas, the importance of the preoperative histological diagnosis has been increasingly emphasized as different therapeutic regimes have been established for different sarcoma types”^7^. The standard diagnostic approach is a core needle biopsy or an open incisional biopsy except for superficial lesions < 3cm and the acral areas, which might be considered for excisional biopsies. Although current clinical guidelines indicate CNB to be the preferred technique over IB, disagreements still exist among clinicians and pathologists regarding the diagnostic accuracy of CNB^8–11^.

CNB has several advantages over open biopsies—in short time for diagnosis, in cost-effectiveness, and in number of patient visits necessary. The most often cited limitations of CNB are the ability to access deep-seated masses and the limited amount of sample material^12,13^. The open biopsy has been considered the gold standard for the diagnosis of STSs. However, IB frequently resulted in wound complications, tumor spreading, and inappropriate incisions, even making following surgical resection more difficult^14,15^.

## Patients and methods

The study was approved by the ethics committee of the Medical University of Vienna, Vienna, Austria (approval number 1345/2020) in accordance with the Helsinki Declaration.

Given the retrospective design of this study, the ethics committee of the Medical University of Vienna waived the requirement for individual informed consent.

### Patient selection

**Figure 1:**
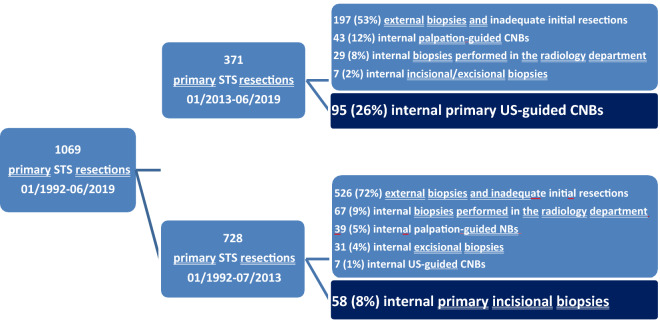
Elimination of patients.

From our institution’s tumor registry, patients who underwent soft tissue sarcoma resection between 01/1992 and 06/2019 were reviewed and classified into two groups according to primary in house biopsy techniques (CNB n = 95, IB n = 58) (Fig. 1). Open biopsy used to be the standard for evaluation of soft tissue tumors for many years. Nevertheless, since 2013 there has been a clear shift in our center towards CNB as the primary biopsy technique. IB has rather been reserved for special indications and cases of insufficient sampling by CNB. Excluded from this study were any cases of bony tissues, well differentiated liposarcoma, subsequent CNBs and IBs, all excisional biopsies, ultrasound-guided CNBs performed in the radiology department and CNBs not performed under US-guidance.

### CNB group characteristics

The institution’s tumor registry provided 95 patients (m 47, f 48; average age at the biopsy: 60 [range 12–87] years) who had between 01/2013 and 06/2019 been treated by soft tissue sarcoma resection and in whom primary ultrasound-guided CNBs were performed at the Department of Orthopedics to diagnose the tumors. In twenty-four patients the final surgical specimens were liposarcoma (17 myxoid, 4 pleomorphic and 3 dedifferentiated), 30 myxofibrosarcoma, 21 undifferentiated pleomorphic sarcoma, 6 synovial sarcoma, 6 leiomyosarcoma and in the remaining 8 patients different STS entities. Sarcoma location of 45 patients was in the thigh, 9 in the lower leg, 9 in the shoulder area, 9 in the upper arm, 9 in the gluteal region, 5 in the thoracic region and the remaining 9 in different body regions (Table 1).

**Table 1:** Diagnostic accuracy in the assessment of dignity.

	Final specimens CNB group	CNB matches	Final specimens IB group	IB matches
**Number of patients**	95	83 (87%)	58	54 (93%)
**Histologic types**				
Myxoid, pleomorphic and dedifferentiated liposarcoma	24	22 (92%)	16	15 (94%)
Undifferentiated pleomorphic sarcoma	21	20 (95%)	18	17 (94%)
Myxofibrosarcoma	30	24 (80%)	3	3
Synovial sarcoma	6	6	4	4
Leiomyosarcoma	6	4	4	4
Rhabdomyosarcoma	3	3	3	3
(Myo)fibrosarcoma	3	2	4	2
Epithelioid sarcoma	–	–	3	3
Malignant peripheral nerve sheath tumor	2	2	–	–
Clear cell sarcoma	–	–	2	2
Alveolar soft part sarcoma	–	–	1	1
**Tumor location**				
Thigh	45	38 (84%)	26	24 (92%)
Knee	2	1	8	8
Lower leg	9	8	10	10
Shoulder/axilla/scapula	9	9	2	1
Upper arm	9	9	2	2
Elbow	–	–	1	1
Forearm	2	2	3	3
Neck	1	1	–	–
Torso	5	4	4	4
Groin	2	2	1	–
Iliac region	2	1	–	–
Gluteal region	9	8	1	1

### IB group characteristics

Fifty-eight patients (m 34, f 24; average age at the biopsy: 44[range 13-83] years) who between 01/1992 and 07/2013 underwent soft tissue sarcoma resection and in whom primary incisional biopsy was performed were identified. The final surgical specimens in 16 patients were liposarcoma (14 myxoid and 2 pleomorphic), 18 undifferentiated pleomorphic sarcoma, 4 synovial sarcoma, 4 leiomyosarcoma, 4 fibrosarcoma and in the remaining 12 cases different STS entities. Twenty-six STSs were located in the thigh, 10 in the lower leg, 8 in the knee area, 4 in the torso, 3 in the forearm and the remaining 7 in different body areas (Table 1).

### Methods

Thorough reviews of medical records including the biopsy, histology and operative reports were performed, and the biopsy results compared with the final surgical specimens. Histological results are based on the pathology reports by experienced bone and soft tissue tumor pathologists at the institution for all patients included in this study.

### Statistical analysis

Statistical analyses were conducted with the Statistical Package for the Social Sciences (SPSS) for Windows (version 21; IBM Co., Endicott, NY, USA). Diagnostic accuracy was defined as the sum of true positive and true negative results divided by the total number of biopsies performed. The significance of differences between frequencies was calculated using Fisher's exact test. A p value of < 0.05 was considered significant.

### Ultrasound technique

Ultrasound was carried out using a GE Healthcare (GE Healthcare, Chicago, IL, USA) LogiqS8, software version Logiq S8: R1.5.4, linear probe:GE Healthcare ML6-15, 15.0Mhz. Needle biopsie was done under sterile conditions with or without coaxial needle with a semi-automatic biopsy needle G14 using “in-plane” technique. Usually, we took 3 to 4 samples.

## Results

### Diagnosing malignancy

CNB correctly identified 87% (83/95) STSs (Table 1). Malignancy was, without any significant differences, reported by CNB in 92% (22/24) liposarcoma, 80% (24/30) myxofibrosarcoma and 95% (20/21) undifferentiated pleomorphic sarcoma cases (Table 1). In three STSs (2%; 2 myxofibrosarcoma and 1 myofibrosarcoma), the dignity was uncertain after CNB. Both myxofibrosarcoma as well as the myofibrosarcoma case were discussed in a multidisciplinary tumor board prior to surgery. Diagnostic accuracy of IB was 93% (54/58). The dignity was uncertain in 2 STSs (3%; 2 myofibrosarcoma), which were both resected due to radiologically suspected malignancy. There was no significant difference in correct malignancy diagnosis rate between the two techniques (p = 0.29).

### Identifying histologic subtype

Correct histologic subtype was by CNB reported in 80% (76/95) of the patients (Table 2). Correctly diagnosed were 80% (24/30) myxofibrosarcoma, 90% (19/21) UPS and 94% (16/17) myxoid liposarcoma (Table 2). There were no significant differences found. Six STSs were by CNB misdiagnosed as other STS subtypes and in 1 patient the subtype could not be identified. Three pleomorphic liposarcoma, 1 dedifferentiated liposarcoma and 1 rhabdomyosarcoma were reported to be UPSs, 1 UPS to be an inflammatory fibrosarcoma and 1 malignant peripheral nerve sheath tumor (MPNST) could only be classified as a nonspecific malignant spindle cell lesion. IB identified correct histologic subtype in 83% (48/58) of the cases. Eighty-nine percent (16/18) UPS and 93% (13/14) myxoid liposarcoma were correctly recognised. Misdiagnosed as other histologic subtype were 5 STSs, and in 1 case the subtype could not be identified.

**Table 2:** Diagnostic accuracy in the assessment of histologic subtype.

	Final specimens CNB group	CNB matches	Final specimens IB group	IB matches
**Number of patients**	95	76 (80%)	58	48 (83%)
**Histologic types**				
Undifferentiated pleomorphic sarcoma	21	19 (90%)	18	16 (89%)
Myxofibrosarcoma	30	24 (80%)	3	1
Myxoid liposarcoma	17	16 (94%)	14	13 (93%)
Synovial sarcoma	6	6	4	3
(Myo)fibrosarcoma	3	2	4	1
Leiomyosarcoma	6	4	4	4
Pleomorphic liposarcoma	4	1	2	2
Rhabdomyosarcoma	3	2	3	2
Dedifferentiated liposarcoma	3	1	–	–
Epithelioid sarcoma	–	–	3	3
Malignant peripheral nerve sheath tumor	2	1	–	–
Clear cell sarcoma	–	–	2	2
Alveolar soft part sarcoma	–	–	1	1

One myxofibrosarcoma was classified as UPS, 1 synovial sarcoma as MPNST, 1 myxofibrosarcoma as myxoid liposarcoma, 1 UPS as leiomyosarcoma, 1 fibrosarcoma as synovial sarcoma and 1 rhabdomyosarcoma could only be classified as a highly malignant sarcoma. No significant difference in correct histologic subtype identification was found between CNB and IB (p = 0.83).

### Grading

An examination of preoperative and postoperative grading concordance was only significant in CNB myxofibrosarcoma cases. Postoperative histological grades were reported in 22 by CNB as myxofibrosarcoma recognised cases. Additionally to histologic subtype, the correct grade was by CNB identified in 73% (16/22) of myxofibrosarcoma.

### Nondiagnostic samples

There were not significantly more nondiagnostic samples in the CNB group than the IB group. The rate of nondiagnostic samples was 9.5% (9/95) in the CNB group and 3% (2/58) in the IB group. There were six samples with necrotic tissue, 2 samples with skeletal muscle and 1 sample with insufficient material in the CNB group. In the IB group was 1 sample with suspected reactive lesion and 1 sample with mostly thrombus material.

### Complications

Patients who underwent CNB showed no complications. In the IB group, 1 male patient (risk factors for a pulmonary embolism: 1. cancer, 2. recent surgery) developed a pulmonary embolism 9 days and 1 female patient (risk factors for pulmonary embolism: 1. cancer, 2. recent surgery, 3. venous insufficiency, 4. smoking, 5. increasing age) 6 days after the surgical procedure. Surgical site infection occurred in 1 patient in whom IB was performed.

The difference in the complication rates between the two techniques was not significant.

## Discussion

“In light of the growing understanding of different biologies and sensitivities of the various STS subtypes, the value of histology in the selection of peri-operative treatments is one of the most intriguing topics under discussion^16^.” In this perspective, discussions about the ideal biopsy techniques are still ongoing. To help solve this diagnostic dilemma, we performed an institutional study regarding the diagnostic performance of CNB and IB in STS. Our results indicate that ultrasound-guided CNB is highly accurate and not inferior to IB in diagnosing the dignity of lesions and histologic subtype in patients with suspected STSs.

The ideal biopsy technique should be characterized by a high diagnostic accuracy, and be simple while minimizing morbidity, limiting potential tumor spread, and avoiding interference with future treatments^8,12^. Siddiqi et al. reported no definable adverse effect on local recurrence is associated with not resecting the CNB tract during definitive operation.

Any influence of a CNB tract resection is likely to be of minor clinical importance^17^. A recent meta-analysis demonstrates fewer complications compared with IB^8^. Similarly, in this study, no complications were seen in patients in whom CNB was performed, whereas pulmonary embolisms occurred in 2 patients who underwent IB. Sarcoma alone is a provoking factor for a pulmonary embolism, recent surgical procedures increase the risk even more^18^. The mobility after incisional biopsies is mostly reduced up to two weeks, whereas there are no limitations after CNB.

The study has several limitations. Our examination of diagnostic accuracy in terms of grading of STSs was limited. Nevertheless, our results were in accordance with the known fact that current grading is not ideal for CNBs. CNB is prone to sampling error with the risk of underestimation of grade^19,20^, and new developments in molecular genetics and targeted therapies question the future of classical grading. It could eventually be replaced by molecular parameters and biomarkers^19,21^.

However, histologic grade is important prognostic factor and indicator of metastatic risk for STSs. Therefore, it should be part of the pathology report and complemented with radiologic parameters when dealing with a needle biopsy^19^.

More than one pathologist was involved in this study and more than one orthopedic surgeon performed the biopsies. The high IB and ultrasound-guided CNB diagnostic performances in our sarcoma center might well be the result of an established workflow between experienced surgeons, radiologists and dedicated sarcoma pathologists. However, without this multidisciplinary setting, there are still numerous cases with incorrect and unplanned open biopsies as the primary biopsy technique performed outside of sarcoma centers. The retrospective data outlined the clear shift in our center toward CNB.

Likewise our study, a meta-analysis published in 2020 reports high CNB accuracy in diagnosing the dignity of lesions and STS histotype in patients with suspected STS^8^. However, two other recent meta-analyses demonstrate a higher diagnostic accuracy for IB compared with CNB^10,11^. The CNB-to-IB ratio was 1.6:1.0 in this study compared to 3–10:1 in the 8 centers included in the meta-analysis carried out by Birgin et al. Moreover, there were no different biopsy techniques and imaging modalities used in the CNB group. All CNBs were performed under US-guidance with a semi-automatic G14 biopsy needle using “in-plane“ technique. Any cases of bony tissues were excluded from this study. All included CNBs and IBs were the primary biopsies, all sequential biopsies were excluded from this study.

## Conclusion

Considering the high diagnostic accuracy rates of both techniques and a significantly higher invasiveness of IB, we recommend ultrasound-guided CNB as the primary technique in patients with suspected STSs. There is always the option to perform IB after CNB in cases of insufficient sampling, but this is not consistently true the other way around. At least 14-gauge needles should be used. To access deep-seated masses in the pelvic region, primary CT- and MRT-guidance should be considered for CNBs.
